# A Real-Time Phase-Locking System for Non-invasive Brain Stimulation

**DOI:** 10.3389/fnins.2018.00877

**Published:** 2018-12-03

**Authors:** Farrokh Mansouri, Peter Fettes, Laura Schulze, Peter Giacobbe, Jose Zariffa, Jonathan Downar

**Affiliations:** ^1^Institute of Biomaterial and Biomedical Engineering, University of Toronto, Toronto, ON, Canada; ^2^Institute of Medical Science, University of Toronto, Toronto, ON, Canada; ^3^Department of Psychiatry, University of Toronto, Toronto, ON, Canada; ^4^Centre for Mental Health, University Health Network, Toronto, ON, Canada; ^5^Harquail Centre for Neuromodulation, Sunnybrook Health Sciences Centre, Toronto, ON, Canada; ^6^Toronto Rehabilitation Institute, University Health Network, Toronto, ON, Canada; ^7^Krembil Research Institute, University Health Network, Toronto, ON, Canada

**Keywords:** closed-loop brain stimulation, synchronized brain stimulation, real-time phase tracking, phase-locked brain stimulation, transcranial electric stimulation

## Abstract

Non-invasive brain stimulation techniques are entering widespread use for the investigation and treatment of a range of neurological and neuropsychiatric disorders. However, most current techniques are ‘open-loop’, without feedback from target brain region activity; this limitation could contribute to heterogeneous effects seen for nominally ‘inhibitory’ and ‘excitatory’ protocols across individuals. More potent and consistent effects may ensue from closed-loop and, in particular, phase-locked brain stimulation. In this work, a closed-loop brain stimulation system is introduced that can analyze EEG data in real-time, provide a forecast of the phase of an underlying brain rhythm of interest, and control pulsed transcranial electromagnetic stimulation to deliver pulses at a specific phase of the target frequency band. The technique was implemented using readily available equipment such as a basic EEG system, a low-cost Arduino board and MATLAB scripts. The phase-locked brain stimulation method was tested in 5 healthy volunteers and its phase-locking performance evaluated at 0, 90, 180, and 270 degree phases in theta and alpha frequency bands. On average phase locking values of 0.55° ± 0.11° and 0.52° ± 0.14° and error angles of 11° ± 11° and 3.3° ± 18° were achieved for theta and alpha stimulation, respectively. Despite the low-cost hardware implementation, signal processing time generated a phase delay of only 3.8° for theta and 57° for alpha stimulation, both readily accommodated in the pulse trigger algorithm. This work lays the methodological steps for achieving phase-locked brain stimulation for brief-pulse transcranial electrical stimulation (tES) and repetitive transcranial magnetic stimulation (rTMS), facilitating further research on the effect of stimulation phase for these techniques.

## Introduction

Non-invasive brain stimulation is entering increasingly widespread use as both a research tool and a clinical intervention for neuropsychiatric disorders. A wide range of non-invasive brain stimulation techniques, such as transcranial magnetic stimulation (TMS) (Hallett, 2000), transcranial electrical stimulation (tES) (Paulus, 2011), and transcranial pulsed ultrasound (TPU) (Tufail et al., 2011), have been developed as ways to modulate brain activity (Polanía et al., 2018). While these methods have demonstrated applications in treating a number of neuropsychiatric disorders (Schulz et al., 2013; Kuo et al., 2014), they are still hampered by several important limitations. One such limitation is the heterogeneity of effect across individuals. For example, nominally ‘excitatory’ or ‘inhibitory’ rTMS protocols can show the opposite-to-expected effect in up to 50% of individuals (Maeda et al., 2000; Hamada et al., 2013). Several hypotheses have been proposed to explain this heterogeneity of effect; prominent among them is a variable degree of synchronization between the applied stimulation and the underlying brain activity when the stimulator is employed in an ‘open-loop’ fashion (Chung et al., 2018). At present, most non-invasive brain stimulation techniques use ‘open-loop,’ i.e., applying stimulation at a set protocol without feedback guidance from the actual activity of the target region. A ‘closed-loop’ system, in contrast, would read the activity of the target region and use this information to guide the parameters of stimulation: for example, the pattern, frequency, phase, or timing of stimulation.

Closed-loop stimulation systems may be considered a worthwhile objective if there is evidence that the effects of stimulation depend on the brain state at the time of stimulation, and indeed there is ample literature support for this proposal. Current models of brain function posit that brain regions operate as integrated networks bound by coherent activity, and task-specific activation of these networks is seen across various brain states (Seager et al., 2002; Park and Friston, 2013; Pessoa, 2014). The state of the brain during the stimulation can change the outcome of the intervention (Silvanto et al., 2008); an elementary example would be the observation that the active motor threshold is substantially lower than the resting motor threshold for stimulation of the primary motor cortex (Hallett, 2007). On a related point, there is mounting evidence that brain stimulation, especially the types that use energies below the threshold for action potential elicitation (e.g., tES), are more consistent in effect when synchronized to the underlying brain activity (Fröhlich and McCormick, 2010; Reato et al., 2013). In one early study, electrical stimulation of hippocampal brain slices showed that when the stimulation was delivered at the peak or the trough of the theta rhythm of tissue, the changes in the evoked potentials were opposite (Hyman et al., 2003). In another example, while TMS effects could vary depending on the phase of the underlying brain network, TMS pulses applied at the peak or trough of the μ-rhythm of the motor cortex have been shown to have opposite plastic effects (Zrenner et al., 2017). Recently, phase-locked stimulation of the sensorimotor cortex in monkeys showed phase specific bidirectional synaptic plasticity (Zanos et al., 2018). Thus, open-loop stimulation, by applying pulses at various phases of the intrinsic brain activity, is proposed to contribute to the observed heterogeneity of effect for stimulation across individuals and across sessions within a given individual. Given these findings, a system enabling phase-locked stimulation could potentially allow more precise control of the direction of effect of the stimulation, as well as a more consistent effect overall, within and across individuals. Such an approach would build upon other efforts to reduce sources of heterogeneity in non-invasive brain stimulation via straightforward, user-friendly, clinically translatable methods (e.g., (Mansouri et al., 2018)).

In tES, the electrical currents applied create a small electric field that can alter the ongoing activity of the brain (Nitsche and Paulus, 2000; Ohn et al., 2008; Utz et al., 2010; Reato et al., 2013). In one example, the frequency of the stimulation matched to the underlying alpha oscillation can modulate the intrinsic alpha rhythms (Vossen et al., 2015). Moreover, frequency- and phase-specific effects of transcranial alternating current stimulation have been shown in a number of specific experiments in motor activity (Guerra et al., 2016; Nakazono et al., 2016), cognition (Polanía et al., 2012) and auditory system (Riecke et al., 2015). However, open-loop implementation of the stimulation techniques in these studies confine them to their experimental settings and inhibit them from being used in a wider scope of applications. Due to the technical challenges of closed-loop brain stimulation, namely real-time implementation of phase tracking algorithms and the presence of stimulation artifacts in the recordings, it has been very difficult to explore the effects of these stimulation techniques in a wide range of models and experimental settings. For example, when applying the sinusoidal alternating current stimuli of transcranial alternating current stimulation (tACS), it becomes difficult to model out the artifact of the stimulation itself in order to recover the original signal from the target brain region, which is needed to recover information about dominant frequency or phase in order to enable closed-loop, phase-locked stimulation. As such, phase-locked tACS or rTMS, although potentially useful in theory for enhancing stimulus potency and consistency, is difficult to accomplish in practice due to challenges in recovering the source signal for closed-loop stimulation.

Here, we describe a novel apparatus for a closed-loop stimulation system that can provide real-time, phase-locked brain stimulation and can be applied with a wide range of neuromodulation techniques. The approach relies on the use of brief pulses of stimulation (as are employed in rTMS, or in this case with brief-pulse tES), such that only a small proportion of the data samples in each cycle are contaminated by stimulus artifact (c.f., Neuropace patent US 6690974B2). The non-contaminated data samples are sufficient for reconstruction of dominant frequency and phase information, and this information can be used in real-time to deliver the stimulation pulses at any desired phase: 0°, 90°, 180°, or 270°. In this work, we have tested our method using pulsed-transcranial electrical stimulation (ptES) (Alon et al., 2012; Berenyi et al., 2012; Morales-Quezada et al., 2015; Vasquez et al., 2016), and lay out the steps essential for implementation of this technique for closed-loop, phase-locked non-invasive brain stimulation. The intended scope of this paper is to describe the technique and provide a preliminary proof-of-concept *in vivo* demonstration. Subsequent work will examine in detail the behavioral effects of phase-locked vs. non-phase-locked stimulation in a larger validation sample.

## Materials and Methods

**FIGURE 1:**
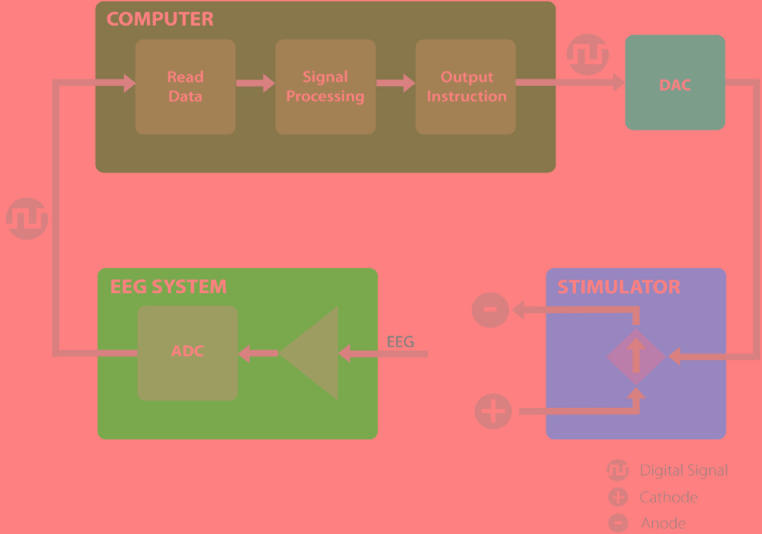
The proposed system, consisting of EEG amplifier (blue) records the EEG signal and transmit the digital data to the computer. The computer (green) receives the EEG recordings and analyze the signal to extract the timing of the stimulation and communicates the instruction through a digital to analog converter to the stimulator. The stimulator (pink), provides the stimulation based on the information received and allows for closed loop brain stimulation. ADC, analog to digital converter; DAC, digital to analog converter.

We have developed a closed-loop brain stimulation technique that is able to read electroencephalography (EEG) data, analyze it in real-time to extract specific phase and frequency information, and control the stimulator based on the phase and frequency of the underlying signal. The system includes an EEG amplifier, MATLAB signal processing scripts and an Arduino interface to control the brain stimulation (Figure 1). In this section we describe all components of this system in detail.

### Real-Time Brain Recording and Preprocessing

A Brain Products V-AMP 16 channel EEG amplifier (Brain Products GmbH, Munich, Germany) was used in this setup. The amplifier was connected to a Windows 7 computer (Intel^®^ Core^TM^ i5-3470 3.2 GHz) via USB. OpenVibe (Renard et al., 2010) was used as an interface to stream data from the amplifier in real-time. The EEG system sampled the data at 2 kHz and downsampled the data to 512 Hz in hardware, while applying the appropriate anti-aliasing filters. The data was buffered and sent 4 samples at a time to the OpenVibe software. The V-AMP amplifier used a 32-bit data resolution with a wide dynamic range. This ensured that the amplifier was not saturated during the recording due to the stimulation and resulting artifacts.

For recording/stimulation sessions, the stimulation electrodes were first placed on the scalp (stimulation: F3, F4 for theta stimulation and O3, O4 for alpha stimulation; recording: Fz for theta stimulation and Oz for alpha stimulation); Ten20 conductive gel (Weaver and Company, Aurora, CO, United States) was applied to the electrodes and the scalp until the impedances of the electrodes were below 5 kΩ. Next, a 16-channel passive-electrode EEG cap (EasyCap GmbH, Germany) was worn by the volunteers on top of the stimulation electrodes and HiCL Abrasive EEG Gel (EasyCap GmbH, Germany) was applied to reduce the impedance of each of the recording electrodes to below 5 kΩ.

### Signal Processing

A MATLAB script was called within OpenVibe every 10ms (100Hz) to analyze the recorded signal. The script was developed to analyze the EEG signal recorded from the EEG channel of interest (Fz for theta recording and Oz for alpha recording) and control the stimulator based on the method presented in our previous work (Mansouri et al., 2017). In summary, this script first removes the stimulation artifact from the recorded EEG; second, it analyzes the EEG to extract the timing for the next stimulation pulse and, finally, it communicates with an Arduino Due board to control the stimulator output in real-time.

#### Artifact Removal

Accurate recovery and analysis of EEG activity occurring simultaneously with a large stimulation artifact is a challenging objective. To date, few methods have been proposed that can remove the electrical stimulation artifacts in short-window recordings (<1 s) in real-time. For stimulation methods such as transcranial alternating current stimulation (tACS), which delivers a continuous sine-wave stimulation pulse, closed-loop stimulation is particularly challenging, as it is difficult to recover the underlying EEG signal accurately, or determine its phase in a given frequency component during active stimulation.

An alternative, workaround approach is to minimize the proportion of EEG samples that are affected by stimulation artifact, by using a tES waveform consisting of short, square-wave pulses rather than continuous sinusoidal stimulation. During brief-pulse stimulation, only a small portion of the EEG samples are contaminated with the stimulation artifact, so that much of the EEG signal (and in particular, its phase in a given frequency component) remains recoverable over a given window of time. For this reason, we used short pulsed stimulation in this work, which enabled us to assess the feasibility of closed-loop, phase-locked tES without the confounding presence of a stimulation artifact (c.f. Neuropace US patent 6690974B2), using readily available and inexpensive components commonly employed in laboratory settings.

The recorded EEG generally has amplitudes smaller than 50 μV, while the stimulation artifacts are orders of magnitude larger (>1 mV). The amplitude of the signal compared with its local median (window size of 20 samples) was used to detect the large artifact (McNames et al., 2004). The empirically selected threshold of 50 μV on the difference between the signal and its median was used to identify the timing of the pulses. Next, the signal contaminated with the artifact was deleted and replaced with an interpolated signal using a spline interpolation method (Waddell et al., 2009).

**FIGURE 2:**
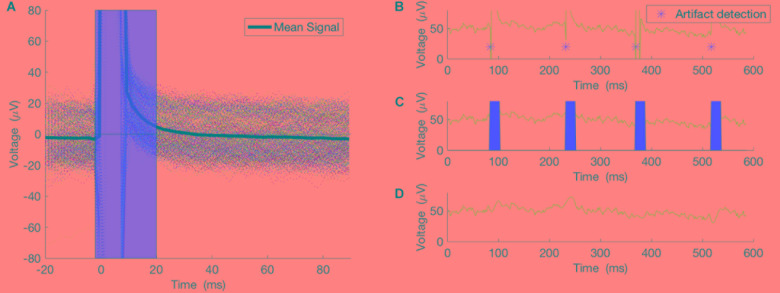
Artifacts from 500 pulses were averaged to study the true effect of the stimulation on the recording. **(A)** The 500 epochs and their average. **(B)** An example of the pulse detector, asterisk showing location of the detected pulses. **(C,D)** Pulses removed and signal interpolated using spline interpolation.

This method worked well only when the duration of the artifact was much smaller than the period of the underlying brain oscillation. In our case, the 5ms pulses generated 20 ms of artifact, while the underlying brain oscillation of interest in the alpha frequency band has a period of ∼100 ms and in the theta frequency band has a period of ∼160 ms, which is much larger than the duration of the artifact itself (Figure 2). For the purposes of this proof-of-concept study, we selected theta (4–8 Hz) and alpha (8–13 Hz) bands for testing this closed-loop phase locking method. To assess the artifact removal method performance, stimulation at arbitrary phase and frequency was applied to study the artifact duration and the artifact detection method. Pulses of 5 ms duration were delivered at random intervals of 100–200 ms. Averaging of 500 epochs of stimulation artifact recordings, time-locked to the onset of the stimulation pulse, was used to assess the lasting effect of the artifact after the onset of the pulse (see Results below).

#### Signal Processing and Frequency and Phase Extraction

As described in our previous work (Mansouri et al., 2017), we first use a segment of the recorded signal and apply an IIR bandpass filter to isolate a frequency band of interest. Based on our previous simulations offline, we used a 10th order elliptical filter, converted the quantized filter to second-order sections, and applied it to the recording.

Next, the Fast Fourier Transform (FFT) of the filtered signal segment was computed. The filtered signal was first zero-padded to increase the bin resolution in the FFT. The FFT bin with maximum power in the frequency range of interest was selected as the dominant frequency and its phase and frequency were used to calculate the timing of the next stimulation pulse. To correct for the small delays in the system or a small phase shift that was introduced by the filter or other components of the signal processing or hardware delays, an empirically calculated correction time was added to the calculated pulse time.

### Real-Time Control of the Stimulator

The timing of the next pulse was communicated to an Arduino Due Microcontroller boar (Arduino) through serial USB communication. First, a serial connection was made through the MATLAB Serial Port Interface (MATLAB version 7.9.0), and at every iteration of the code the timing of the next pulse was communicated to the Arduino Due, which then produced a pulse through its true analog output pin with microsecond resolution. The Arduino analog output is connected to the “remote control” port on the Neuroconn DC-Plus stimulator (Neuroconn Ltd., Ilmenau, Germany). In this system, the stimulator follows the Arduino-generated voltage waveforms and produces a current-controlled output proportional to that voltage.

#### *In vivo* Demonstration

In order to demonstrate the potential applicability of the present technique across different brain regions, different states (resting vs. on-task), and different recording types (ongoing activity vs. evoked potential on-task), we studied both alpha and theta rhythms, both occipital and frontal regions, and both resting and on-task (evoked) brain states. Occipital regions are particularly potent and well-studied generators of resting alpha rhythms, particularly during the resting eyes-closed state, and are thus widely used in studies of EEG alpha activity (Lehmann, 1971; Vossen et al., 2015). Likewise, midline frontal regions are particularly potent and well-studied generators of theta rhythms, particularly during cognitive control tasks, which are likewise widely used in EEG studies of cognition in healthy controls and individuals with illness (Frank et al., 2004, 2005; Cavanagh and Frank, 2014). Such studies commonly employ evoked potentials rather than resting-state activity (Frank et al., 2004, 2005; Cavanagh and Frank, 2014).

The system was tested by providing phase-locked stimulation at theta (4–8 Hz) and alpha (8–13 Hz) in mid-frontal and occipital human brain regions, respectively. The testing was performed during eyes closed EEG for alpha-band testing (5 min). For theta-band testing sessions (5 min), the participants played a computer-based reinforcement learning game (Frank et al., 2004, 2005). In this reinforcement learning game, participants were presented with pairs of Japanese characters and asked to choose one by pressing left or right key on a keyboard, followed by a visual feedback (won or lost) (Frank et al., 2004, 2005). 5 millisecond square-wave monophasic pulses of 2 mA current were delivered at 0, 90, 180, or 270 phase angle. Each stimulation (alpha and theta) was applied for 50 pulses for each of 0^o^, 90^o^, 180^o^ and 270^o^ phase angles. Phase-locking values for each band and phase-angle were computed as below, along with their distributions for each phase and band.

#### Participants

Participants were 5 healthy volunteers (3 male, 2 female, ages 27–30, mean age 28.0 ± 1.6 (mean ± SD), 2 left-handed). All recruitment, informed consent, and experimental procedures were approved by the Research Ethics Board of the University Health Network (UHN REB 16-5270) in accordance with the principles of the Declaration of Helsinki.

### Analysis

To measure the phase of the underlying brain oscillation during the stimulation, the stimulation artifact was first removed and the signal interpolated using the methods described earlier. Then, the bandpass filter was applied. The phase of the EEG was measured as the angle of the Hilbert transformation during the stimulation pulse. We use polar histogram plots to visualize the phase of the stimulation compared to the phase of the underlying brain oscillations. Further, we calculated Phase Locking Value (PLV) (Equation 1) and the mean angle of the phase (Equation 2). PLV is a value between 0 and 1; higher PLV shows better phase locking. In these equations, φ is the phase angle of the underlying EEG frequency component of interest during the stimulation pulse, and N is the number of pulses used to calculate PLV and mean angle. All values in this manuscript are reported as mean ± standard deviation.

Equation 1:

$$\mathrm{PLV}=\left\|\frac{1}{N}\sum_{n=1}^{N}e^{\varphi_{n}i}\right\|$$

Equation 2:

$$Mean\;Angle=∠\left(\frac{1}{N}\sum_{n=1}^{N}e^{\varphi_{n}i}\right)$$

## Results

### Artifact Removal

As noted above, stimulation at arbitrary phase and frequency was applied to study the artifact duration and the artifact detection method. Pulses of 5ms duration were delivered at random intervals of 100–200 ms. Averaging 500 epochs of stimulation artifact recordings, time-locked to the onset of the stimulation pulse, illustrates the lasting effect of the artifact after the onset of the pulse (Figure 2A). Thus, our artifact removal method is set to remove 20 ms of the recording and interpolate the signal for that duration (Figures 2B–D).

### System Timing Corrections

**FIGURE 3:**
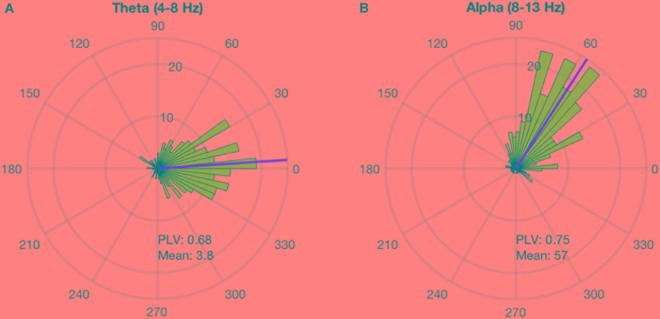
Phase-locking delay for theta and alpha sessions. The phase-locked stimulation without any phase correction shows a constant delay. This delay is calculated for each stimulation frequency. **(A)** The stimulation at theta band for 300 pulses showed that the system has a delay of 3.8 degrees and PLV of 0.68. **(B)** The stimulation at alpha for 300 pulses showed that the system has a delay of 57 degrees and PLV of 0.68.

Alpha and theta stimulation were conducted on a healthy volunteer to test the system delays and calculate the angle corrections for each of the stimulations. The stimulation was applied for 300 pulses and the analysis showed that alpha stimulation is delayed 57 degrees, while theta stimulation is delayed 3.8 degrees (Figure 3). A PLV of 0.68 was found for theta stimulation, while alpha stimulation achieved a PLV of 0.75. For the subsequent testing work that followed these measurements, a timing correction was implemented in the system to compensate for the delays.

### *In vivo* Demonstration

Both alpha and theta stimulation sessions were applied successfully in all 5 participants. The volunteers reported no pain from the stimulation and there were no phosphene experiences reported. A minor tingling sensation was reported by 3 of the participants. All stimulation electrodes and the recording electrodes had impedances of smaller than 5 kΩ throughout the experiment.

**FIGURE 4:**
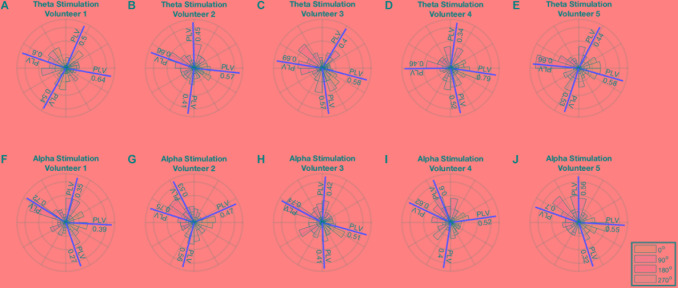
**(A–E)** Polar histogram showing performance of theta stimulation in theta band for 4 different phase angles. **(F–J)** Polar histogram showing performance of theta stimulation in theta band for 4 different phase angles. The mean angle for each stimulation is shown by the red line and the PLV value is reported on the red line.

A minimum of 0.34 PLV and maximum of 0.79 PLV and average of 0.55 ± 0.11 was achieved for theta stimulation. A minimum of 0.27 PLV and maximum of 0.75 PLV and average of 0.52 ± 0.14 was achieved for alpha stimulation. The average error in the angles were 11^o^ ± 11^o^ for theta and 3.3^o^ ± 18^o^ for alpha stimulation. Thus, the distribution of phase angles of stimulation for each of the 4 phase angles remained well within in their particular quadrant (i.e., with standard deviations 2.5–4 times smaller than ± 45° in each instance) (Figure 4).

## Discussion

Endogenous brain activity during stimulation is a key factor determining the effect of the stimulation (Herrmann et al., 2013; Fröhlich, 2014). As a result, conventional ‘open-loop’ brain stimulation techniques such as rTMS, tDCS, or tACS that do not make efforts to synchronize their activity to the endogenous activity of the target brain region risk the possibility of an unwelcome heterogeneity in the magnitude, or even direction, of their effect. For example, nominally ‘inhibitory’ rTMS protocols such as 1 Hz or continuous theta-burst stimulation show the opposite effect (i.e., facilitation) in up to 50% of individuals; conversely, inhibitory effects are seen in a substantial proportion of subjects for nominally ‘excitatory’ rTMS protocols such as 10 Hz or intermittent theta-burst stimulation (Maeda et al., 2000; Hamada et al., 2013). Regarding one possible factor behind this heterogeneity, recent results indicate that phase-locking the stimulation in- or out-of-phase to the endogenous activity of the target can render a given protocol either inhibitory or excitatory (Zrenner et al., 2017), highlighting the importance of phase-locking specifically, and of the need for closed-loop brain stimulation methods in general. Closed-loop brain stimulation thus has the potential to overcome a major current hindrance to the effectiveness of neuromodulation techniques, by improving the potency and consistency of effect (Karabanov et al., 2016).

Here we have implemented a closed-loop brain stimulation that is suitable for operation in real time, targeting specific EEG bands of common interest in research and clinical settings: the theta and alpha EEG frequency bands. The methods introduced in this work demonstrate a functioning apparatus for the development and testing of stimulation phase effects on the underlying brain oscillation. The phase measurement of the stimulation from the recording would be unlikely to arise from a pick-up of the stimulation artifact, because this would mean that the phase measured would always be the same, and thus would not allow phase locking at 4 different phases, as illustrated in Figure 4. Using a simple apparatus whose components offer the advantages of low cost and wide availability, we developed a system that can analyze the EEG using our previously published algorithm (Mansouri et al., 2017) and provide real-time control of the stimulation. In this study, the analysis of the phase relies on a single channel recorded at the scalp location of interest. The technique is for this reason compatible with larger, higher-density arrays, but also works successfully in more limited arrays, as the present study illustrates. Using larger EEG systems would not affect performance of the proposed technique given that a single channel is used for input.

One major hurdle for closed-loop brain stimulation is the artifact introduced by the stimulator itself. Yet, despite extensive and inventive work to solve this problem (Kohli and Casson, 2015; Dowsett and Herrmann, 2016; Noury et al., 2016; Noury and Siegel, 2017, 2018; Kasten et al., 2018), to our knowledge there is still no effective solution for this issue that leverages inexpensive, routinely available components suitable for routine clinical/translational use. In the present work, we suggest that a viable workaround is to employ brief stimulation pulses (as is the case with rTMS, or brief-pulse tES) so as to minimize the proportion of samples affected by artifacts, and avoid using the segments of the recording that have been contaminated with the artifact. We used pulsed stimulation and showed that in our recording system, each 5 ms pulse of stimulation contaminates at most ∼20 ms of the recording. Considering that the target frequency bands have periods much larger than 20 ms, it is possible to use the recordings with missing 20 ms sections and still determine the phase and frequency of the underlying brain activity with good accuracy.

In this work, we have tested this system on theta and alpha bands, noting that these bands are often of interest in EEG and tACS studies (Jaušovec and Jaušovec, 2014; Pahor and Jaušovec, 2014; Vossen et al., 2015; Vosskuhl et al., 2015) and that they have long enough wavelengths to enable successful extraction of phase and frequency information even after artifact removal. Another feature of the present system lies in circumventing the effects of the artifact by applying the stimulation in a discontinuous mode. By introducing a delay between the stimulation pulses and providing enough recording time between the pulses, the artifact of the first stimulation pulse will not be present in the recordings used for estimating the timing of the next pulse. Of note, novel non-electromagnetic stimulation modalities, such as focused ultrasound (e.g., TPU), can be applied without contaminating the EEG signal with any artifact at all. Such forms of neuromodulation will perhaps eventually allow us to circumvent the problem of electromagnetic stimulation-induced artifacts altogether.

It is worth noting that signal processing components, such as the hardware filters used in EEG recording instruments, and the software filters applied to the signal during processing, can introduce frequency dependent delays to the recordings. Additionally, computation time delays and hardware delays communicating the stimulation instructions to the stimulator are inevitable. In this work, we addressed this issue by measuring the phase delay introduced by all the components of the combined system, and then applying a phase correction to achieve the desired stimulation phase. The execution time of the MATLAB code in this study was ∼1 ms. This delay is proximately constant and adjusted for through empirically calculated phase correction. Considering that the delay in the MATLAB computation time is two orders of magnitude smaller than the period of the oscillation of interest (∼100–200 ms), small changes in the computation time do not significantly affect the accuracy of the phase locking method. Using faster hardware and more optimized signal processing techniques can potentially reduce these delays, but not remove them entirely; thus, the phase delay lag should ideally be addressed via a phase-adjustment correction factor that can help improve the overall phase-locking performance of the system.

It is also important to note that the applicability of our approach is not limited to pulsed electrical stimulation; rather, it is also suitable for a number of pulsed stimulation techniques including rTMS, tES and TPU. The present method is applicable to any stimulation technique in which the stimulation pulses are brief compared to the overall period of the endogenous waveform of interest. For rTMS pulses, which are <1 ms in duration, the present method is suitable not only for theta- and alpha-band locking, but potentially also for higher-frequency bands of interest such as the beta or gamma bands. For low-field electromagnetic stimulation (Rohan et al., 2014) or transcranial pulsed electromagnetic field stimulation (Martiny et al., 2010),the stimulation pulses are likewise much briefer than the period of the EEG bands of interest, and the present method may be applicable to enable phase-locked stimulation. Since the effects of phase and timing for these stimulation modalities are still only just beginning to be explored, the approach presented here may enable exploration of previously neglected dimensions of the parameter space for non-invasive brain stimulation protocols.

An important limitation of the present work, and a topic for future study, concerns the actual effects of this short-pulse (5 ms) ptES waveform on brain activity and behavior across a larger sample of individuals sufficient to generate an estimate of the distribution of performances in the general population. A future study will address this larger aim; the more limited purpose of the present study was to describe the technique and apparatus, and to demonstrate its successful use as proof of concept. Although in this study we were able to introduce a closed-loop brain stimulation method in a relatively small group, its effect on the target brain region’s electrical oscillations themselves remains uncharacterized. In addition, the effect of the ptES on behavioral measures of brain function (e.g., working memory performance, or reaction times on a cued-response task) remains to be characterized in more detail. The available evidence to date suggests that short pulses of transcranial electrical stimulation, similar to those used in the present study, are indeed capable of modulating brain activity and behavior. For example, transcranial pulsed electrical stimulation can modulate spike and waves of seizures in an epileptic rat model (Berenyi et al., 2012). Furthermore, Alon et al. (2012) have shown acute improvement in gait and balance recovery in a Parkinson’s disease population using this type of stimulation. Furthermore, pulsed stimulation has been recently shown to alter cognitive performance and heart rate variability (HRV) across a range of cognitive tasks (Morales-Quezada et al., 2015). In addition, Vasquez et al. (2016) showed pulsed stimulation significantly increases alpha and theta coherence in frontal regions. Nonetheless, the specific effects of in- and out-of-phase stimulation for the brief, 5 ms, 2 mA pulses of the present study on brain activity and behavior remain to be characterized in future studies.

In conclusion, there is mounting evidence that brain stimulation at different phases of the underlying brain oscillation can have quite different effects on brain activity and its behavioral sequelae (Polanía et al., 2012; Brittain et al., 2013; Riecke et al., 2015; Guerra et al., 2016; Nakazono et al., 2016). At the same time, research on the effects of phase-locked stimulation has been hampered by the challenges of recovering accurate EEG signal from a target brain region, while simultaneously stimulating that same region with sinusoidal waveforms, such as those used in tACS. The workaround of the present study is to apply brief, square-wave tES pulses, such that the artifact is limited to only a small proportion of samples and the phase information can still be recovered from the EEG signal with good accuracy. A system composed of readily available, off-the-shelf components can recover the phase information using this method, and can apply it in real time to control pulse timing, while compensating for processing lags to maintain phase-locked stimulation in both the alpha and the theta bands. This work therefore presents a straightforward and inexpensive, yet viable, approach to achieving closed-loop, phase-locked brain stimulation. With further validation, this method may allow a systematic assessment of the effect of phase-locking on the neurobiological and behavioral effects of ptES in both healthy volunteers and patient populations. If phase-locking can indeed reduce the heterogeneity of effect for non-invasive brain stimulation, then there may be potential for marked increases in the efficacy of tES, rTMS, and other brain stimulation techniques in the years to come.

## Author Contributions

All authors listed have made a substantial, direct and intellectual contribution to the work, and approved it for publication.

## Conflict of Interest Statement

The authors declare that the research was conducted in the absence of any commercial or financial relationships that could be construed as a potential conflict of interest.
